# The role of JAK3 and TEC family kinases in vitiligo pathogenesis

**DOI:** 10.3389/fimmu.2026.1662245

**Published:** 2026-03-16

**Authors:** Thierry Passeron, Julien Seneschal, Mauro Picardo, Leihong Xiang, Atsushi Tanemura, Aaron Winkler, Jean-Baptiste Telliez, Roni Adiri

**Affiliations:** 1 University Côte d’Azur, Centre Hospitalier Universitaire Nice, Department of Dermatology, Nice, France; 2 University Côte d’Azur, Institut national de la santé et de la recherche médicale (INSERM), U1065, C3M, Nice, France; 3 Centre Hospitalier Universitaire (CHU) de Bordeaux, Department of Dermatology and Pediatric Dermatology, National Reference Center for Rare Skin Diseases, Hôpital Saint-André, UMR 5164, Bordeaux, France; 4 University Bordeaux, French National Center for Scientific Research (CNRS), Immuno ConcEpT, UMR 5164, Bordeaux, France; 5 Istituto Dermopatico Immacolata, Scientific Hospitalization and Treatment Institute (IRCCS), Rome, Italy; 6 Department of Dermatology, Huashan Hospital, Fudan University, Shanghai, China; 7 Department of Dermatology Integrated Medicine, Osaka University Graduate School of Medicine, Osaka, Japan; 8 Inflammation and Immunology Research Unit, Pfizer Inc., Cambridge, MA, United States; 9 Pfizer Pharmaceutical Israel LTD, Herzliya Pituach, Israel

**Keywords:** autoimmune disorder, cytokine, disease pathogenesis, JAK3 (Janus kinase 3), JAK-STAT signaling pathway, targeted therapy, TEC family kinases, vitiligo

## Abstract

Vitiligo is an autoimmune disease characterized by the loss of skin pigmentation due to the loss of melanocytes. The pathogenesis of vitiligo is complex, involving multiple genetic factors, environmental triggers, oxidative stress, and autoimmunity against melanocytes. Stressed melanocytes release damage-associated molecular patterns, which trigger increased activation of antigen presenting cells, leading to maturation and activation of CD8^+^ T-cells that respond to auto-melanocyte-specific antigens. Once recruited to melanocytes, cytotoxic CD8^+^ and CD4^+^ T-cells produce cytokines, including primarily the type 1 cytokine IFN-γ, but also IL-2, IL-15, and type 2-related cytokines. Cytokines bind to fibroblasts, melanocytes, and keratinocytes to induce a positive feedback loop of immune cell recruitment to lesions, immune cell activation, and melanocyte apoptosis. The JAK/STAT pathway and TEC family kinase signaling play key roles in vitiligo pathogenesis through chemokine production, reduction of melanocyte adhesion, and immune cell activation and disease maintenance. This review summarizes recent key advances in understanding how these pathways impact vitiligo pathogenesis and details the emergence of new targeted therapies for the treatment of vitiligo.

## 1 Introduction

Vitiligo is an autoimmune disease characterized by depigmentation of the skin, hair, or both. Vitiligo diagnosed by a physician or dermatologist has an estimated worldwide prevalence of 0.36-2% (95% CI: 0.24%–0.54%) (1–4). Patients with vitiligo experience greater incidences of comorbidities such as anxiety and depression compared with those without vitiligo (2, 5–10). As a global disease, vitiligo affects patients of all sexes, races, and ethnicities (1, 2).

The majority of vitiligo cases (84%-95%) are classified as “nonsegmental vitiligo” (NSV); NSV confers symmetrical patches extending to both sides of the body, and an unpredictable disease course (1, 3). Common comorbidities for individuals with vitiligo include autoimmune diseases such as thyroid disease (11, 12), alopecia areata (13), atopic dermatitis (14), type I diabetes (12, 15), and psoriasis (16).

Vitiligo has a complex pathogenesis and historically has had few targeted treatment options. Collectively, this review will provide an overview of vitiligo’s pathogenesis and therapeutic landscape with a focus on two pathways that are a current area of therapeutic focus. The specific roles the Janus kinase/signal transducer and activator of transcription (JAK/STAT) pathway and the tyrosine kinase expressed in hepatocellular carcinoma (TEC) family kinase member pathway play in immune cell signaling will be highlighted to describe the current landscape of targeted treatments in development. We will discuss therapies targeting the JAK/STAT and TEC family kinases as targets that are under development, including a review of currently approved targeted treatments and those in various stages of development. Finally, we will highlight key evidence gaps and areas requiring further investigation as vitiligo treatment enters a new era.

## 2 Vitiligo pathogenesis overview

Vitiligo pathogenesis is a multifactorial process involving genetic factors, environmental triggers, oxidative stress, and autoimmunity against melanocytes (17). Large-scale genomic studies have identified allelic variations in genes associated with immune regulation and melanogenesis as significant contributors to vitiligo (16, 18–21). In this section, we will discuss the various reasons for pathogenesis, as well as introduce key cytokines and chemokines that result in melanocyte apoptosis.

Environmental triggers, such as oxidative stress, also play a role in vitiligo pathogenesis. Melanocytes in patients with vitiligo may be more susceptible to oxidative stress. Due to various events such as chronic friction and impaired mitochondrial and melanosome functioning leading to redox imbalances, reactive oxygen species (ROS) can accumulate in the skin and melanocytes, leading stressed melanocytes to release damage-associated molecular patterns (1, 22, 23). These DAMPs are then delivered to nearby dendritic cells. Dendritic cells uptake and process antigens into peptides that are then loaded onto major-histocompatibility class 1 (MHC-I) or MHC class 2 (MHC-II) molecules and presented at the cell surface for T cell recognition, which trigger the differentiation of naive T-cells into cytotoxic CD8^+^ T-cells or CD4^+^ T-cells, respectively (24). This recognition is handled by T-cell receptors (TCR). Pathogen-associated-molecular patterns (PAMPs) can also activate the innate immune system, although the correlation between PAMPs and vitiligo is not well understood. Viral infections may also induce ROS production (23), and a link between changes in gut and skin microbiomes in patients with vitiligo and mitochondrial damage has recently been demonstrated (25). Mitochondrial oxidative stress has also been linked to the release of mitochondrial DNA (22). This triggers the activation of TANK-binding kinase 1 downstream of the cyclic GMP‐AMP synthase‐stimulator of interferon genes pathway, and the induction of pro‐inflammatory cytokines and chemokines responsible for the attraction of CD8^+^ cells and the initiation of an auto‐immune response against melanocytes (22).

In the skin surrounding vitiligo lesions, activated dendritic cells and CD8^+^ T-cells produce cytokines (**Table 1**) such as interferon (IFN)-γ, tumor necrosis factor (TNF)-α, interleukin (IL)-12, and IL-13, which induce keratinocytes, fibroblasts, and melanocytes to produce chemokines, such as C-X-C motif chemokine ligand (CXCL)9, CXCL10, CXCL11, C-C motif chemokine ligand (CCL)5, and CCL18 (26–31). In response to IFN-γ signaling, keratinocytes secrete CXCL9 and CXCL10, which coordinate T-cell activation and recruitment (26). IFN-γ also stimulates fibroblasts to produce CXCL10, which also contributes to CD8^+^ T-cell recruitment to melanocytes. However, fibroblasts can exhibit differential responses to IFN-γ stimulation, resulting in variable vulnerability in certain locations to lesion development (32). C-X-C motif chemokine receptor (CXCR)3, once activated by IFN-γ signaling, also plays a role in maintaining pre-existing vitiligo lesions, and autoreactive T-cell migration (27). The positive feedback loop between IFN-γ, CXCL9/10, and CXCR3 is a key factor in ongoing inflammation and autoimmune response during vitiligo pathogenesis (33). CCL5 is upregulated by both IFN-γ and TNF-α, and both recruits antigen-presenting cells (APCs) such as dendritic cells, B-cells, and macrophages and induces CD8^+^ T-cells to attract further regulatory T-cells to the sites of lesions (30, 34–36). CCL18 meanwhile, is increased in melanocytes by IFN-γ and IL-13, inducing a T helper 2 (Th2) response (30).

**Table 1:** Cytokines and chemokines affecting vitiligo pathogenesis.

Cytokine	Family	Secreted by	Active on	Role in vitiligo
IL-2 (27, 46, 48, 63–66, 78, 79, 81–83)	Interleukin Type I	CD8^+^, CD4^+^ T-cells	CD49a^+^/CD8^+^ tissue-resident memory T cells	Disease activity maintenance and melanocyte apoptosis
IL-4 (64–66)	Interleukin Type I	Naïve helper T-cells	Th2 cells	Elevated type 2 immune response
IL-7 (42, 58, 64–66)	Interleukin Type I	Keratinocytes	CD49a^+^/CD8^+^ tissue-resident memory T cells	Disease activity maintenance and melanocyte apoptosis
IL-9 (57, 64–66)	Interleukin Type I	CD4^+^ T cells	Melanocytes	Decreased ROS
IL-12 (75, 76)	Interleukin Type I	Dendritic cells	Th1 cells, cytotoxic T-cells	Elevated type 1 immune response
IL-13 (30)	Interleukin Type I	CD8^+^ T-cells	Th2 cells	Elevated type 2 immune response
IL-15 (27, 42, 46–48, 58, 63–66, 78, 79, 84)	Interleukin Type I	CD8^+^, CD4^+^ T-cells, keratinocytes	CD49a^+^/CD8^+^ tissue-resident memory T cells	Disease activity maintenance and melanocyte apoptosis
IL-21 (51, 64–66)	Interleukin Type I	CD4^+^ T-cells	Cytotoxic T cells	Increased immune activity
IL-23 (48, 54–56)	Interleukin Type I	Dendritic cells	Th17 cells	Elevated type 17 immune response
IL-33 (54)	Interleukin Type I	Stressed cells	Th2 cells	Elevated type 2 immune response
IFN-γ (27, 30, 34, 46, 63, 78, 79, 92)	Interferon Type II	CD8^+^, CD4^+^ T-cells, autoreactive Trm cells	Keratinocytes, fibroblasts, melanocytes	Chemokine production leading to immune cell activation and melanocyte-reactive T-cell recruitment, melanocyte detachment and apoptosis
IL-22 (54)	Interleukin Type II	T-cells	Th22 cells	Elevated type 22 immune response
Il-17 (51–53)	Interleukin Type III	CD8^+^, CD4^+^ T-cells	Th17 cells	Elevated type 17 immune response
TGF-β (42, 58)	TGF- β	Dendritic cells	CD49a^+^/CD8^+^ tissue-resident memory T cells	Disease activity maintenance and melanocyte apoptosis
TNF-α (30, 34, 46, 109)	TNF	CD8^+^, CD4^+^ T-cells, autoreactive Trm cells	Fibroblasts, keratinocytes	Chemokine production leading to immune cell activation and melanocyte-reactive T-cell recruitment, melanocyte detachment and apoptosis
Chemokine	Type	Secreted by	Active on	Role in vitiligo
CCL5 (26–31, 34)	Chemokine ligand	Keratinocytes, fibroblasts, melanocytes	CD8^+^ T-cells	Recruitment of regulatory T-cells
CCL18 (26–31)	Chemokine ligand	Keratinocytes, fibroblasts, melanocytes	Melanocytes, CD8^+^ T-cells	Elevated type 2 immune response
CXCL9 (26–31, 44)	Chemokine ligand	Keratinocytes, fibroblasts, melanocytes	T-cells, melanocytes	T-cell activation and recruitment to melanocytes
CXCL10 (26–31, 44)	Chemokine ligand	Keratinocytes, fibroblasts, melanocytes	T-cells, melanocytes	T-cell activation and recruitment to melanocytes
CXCL11 (26–31, 44)	Chemokine ligand	Keratinocytes, fibroblasts, melanocytes	Melanocytes	Induce apoptosis and T-cell activation
CXCR3 (27, 44)	Chemokine receptor	CD8^+^ T-cells	CD8^+^ T-cells	Disease maintenance, T-cell migration, apoptosis

CCL, C-C motif chemokine ligand; CXCR, C-X-C motif chemokine receptor; CXCL, C-X-C motif chemokine ligand; IFN, interferon; IL, interleukin; TGF, transforming growth factor; ThX, T helper type X; TNF, tumor necrosis factor.

**Figure 1:**
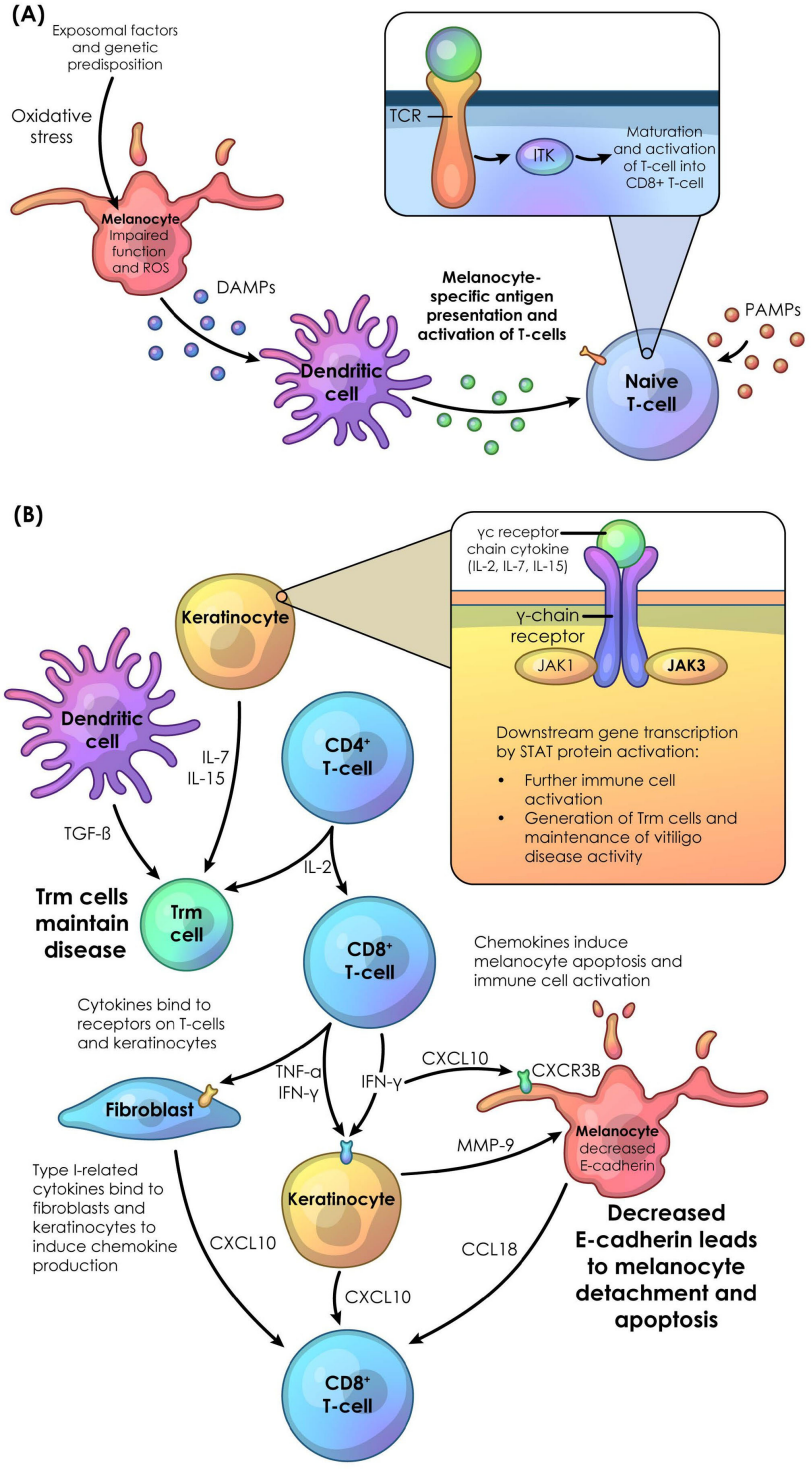
Immune dysregulation leading to vitiligo pathogenesis. **(A)** In vitiligo, many factors including genetic predisposition, and environmental triggers can lead to an increase in oxidative stress and ROS in melanocytes, leading to impaired function. Stressed melanocytes release DAMPs, which trigger dendritic cells to induce activation of naive T-cells through uptake of DAMPs and subsequent production of antigenic peptide:MHC-I complexes. These complexes interact with TCR, and activation of these receptors leads to recruitment of TEC family kinases such as ITK, which instigates a signaling cascade resulting in maturation and activation of CD8^+^ T-cells. PAMPs can also activate the innate immune system and may induce vitiligo pathogenesis. **(B)** CD4^+^ T-cells, through IL-2, stimulate T-cell proliferation. CD8^+^ T-cells, through TNF-α and IFN-γ, bind to fibroblasts, melanocytes, and keratinocytes to induce chemokines such as CXCL10, resulting in a positive feedback loop of immune cell recruitment to lesions, immune cell activation, and melanocyte apoptosis. Keratinocytes are also stimulated to produce MMP-9, which decreases melanocyte E-cadherin, leading to melanocyte detachment and further apoptosis. CD4^+^ T-cells through IL-2 signaling, keratinocytes through IL-7 and IL-15 signaling, and dendritic cells through TGF-β signaling, induce the creation, activation, and maintenance of tissue-resident memory T cells (Trm cells). Trm cells maintain disease even after inflammation is reduced. DAMP, damaged-related molecular pattern; IL, interleukin; ITK, interleukin-2-inducible T-cell kinase; JAK, Janus kinase; MHC, major-histocompatibility complex; PAMP, pathogen-related molecular pattern; ROS, reactive oxidative stress; TCR, T-cell receptor; Trm cell, tissue-resident memory T cell.

These chemokines further induce immune cell activation and recruit T-cells to the site of stress (**Figure 1**) (37–42). Chemokines also decrease melanocyte adhesion and down regulate genes related to pigmentation (26–30, 43). CXCL9, CXCL10, and CXCL11 activate CXCR3B on the surface of melanocytes, which induces apoptosis and further T-cell activation as well as further expression of *IFN-γ* (44). High expression of *CXCR3B* on the surface of melanocytes has been observed in patients with vitiligo, which may potentially prime the melanocytes for apoptosis, and following cell death, trigger further melanocyte auto-immunity (44).

Type 1 cytokines, such as IFN-γ, and Type 2 cytokines also play an important role in melanocyte apoptosis. IFN-γ can induce keratinocytes to produce Matrix metalloproteinase (MMP)-9, leading to melanocyte detachment through a decrease in melanocyte E-cadherin (28).

In patients with vitiligo, high levels of IFN-γ levels are sustained in part due to secretion by other members of the innate immune system, including natural killer (NK) cells and type 1 innate lymphoid cells (23, 45).

Cytotoxic CD8^+^ and CD4^+^ T-cells, once recruited to melanocytes, produce cytokines that induce detachment and apoptosis, including primarily IFN-γ, but also TNF-α, IL-2, and IL-15 (27, 46–50). Additionally, type 2-related cytokines like IL-13 and type 3-related cytokines such as IL-17 and IL-21 have been observed in affected lesions (30, 51). However, anti-IL-17 therapy has been shown to be ineffective in treating vitiligo, and in some cases may induce onset of disease (52, 53).

Other cytokines such as IL-22, IL-23, and IL-33 have been shown to be elevated in patients with vitiligo compared with healthy controls (54). The role of IL-23 in vitiligo pathogenesis is unclear. A small number of case studies have indicated that anti-IL-23 therapy may induce repigmentation (55), while another case study reported that anti-IL-23 therapy induced vitiligo (56).

Conversely, IL-9 signaling through CD4^+^ T-cells may reduce oxidative stress in melanocytes (57).

Additionally, IL-7 and IL-15, secreted by keratinocytes, and transforming growth factor-β, secreted by dendritic cells in the skin, play roles in activating and maintaining tissue-resident memory T cells (42, 58). In patients with vitiligo, autoreactive tissue-resident memory T cells secrete IFN-γ and TNF-α, as well as effector proteins perforin and granzyme B, resulting in further immune activation to melanocytes (46). In lesional skin, tissue-resident memory T cells maintain high levels of IFN-γ, thus continuing the positive feedback loop of IFN-γ and the CXCL axis. These cells can persist in the skin even after inflammation is reduced, potentially contributing to disease relapse after treatment discontinuation in lesional skin (59–61). In non-lesional skin, tissue-resident memory T cells display somewhat heightened activation, which may induce disease activation in new locations distant to the original lesions (34).

## 3 Role of JAK3 and TEC family kinase signaling in vitiligo pathogenesis

The various signaling molecules discussed above signal using the JAK/STAT and TEC family kinase pathways. In this section, we will discuss the impact these pathways have on vitiligo pathogenesis in more specific detail.

### 3.1 The JAK/STAT pathway

The JAK/STAT pathway is an intracellular signal transduction pathway expressed in all cell types. It is responsible for broad biological processes, including proliferation, differentiation, apoptosis, and immune system regulation through cytokine-mediated signaling, with the JAK family consisting of four main members: JAK1, JAK2, JAK3, and tyrosine kinase 2 (TYK2) (62, 63). Upon activation by an associated receptor chain cytokine, members of the JAK family become phosphorylated, initiating a signaling cascade that results in the translocation of phosphorylated STAT protein dimers to the nucleus and transcriptional changes that further activate the immune response (64–66).

*JAK1*, *JAK2*, and *TYK2* are expressed in all tissues (64–66). JAK1 can be phosphorylated by the γc receptors (such as IL-2, IL-7, and IL-15 receptors), class II cytokine receptors (such as IFN-α/β/γ receptors), and gp130 subunit receptors (such as IL-16 receptor and leukemia inhibitory factor receptor) (67, 68). JAK2 can be phosphorylated by class II cytokine and gp130 subunit receptors, but also participates in IL-3 receptor signaling (69). Knockout of either *Jak1* or *Jak2* is embryonically lethal in mice (67, 70). TYK2 participates in IFN-α/β, IL-6, IL-10, IL-12, IL-13, and IL-23 signaling (66, 71). Unlike mice lacking *Jak1* or *Jak2*, *Tyk2* deficiency is not embryonically lethal; these mice display dysfunction in T helper type 1 (Th1) and Th2 cell signaling instead (72). Humans lacking TYK2 display severe allergies (73). In vitiligo, JAK1 and 2 are activated directly by IFN-γ, leading to CD8+ T cell recruitment (63, 74). TYK2, through regulation of Th1 cells and IL-12, can also create an elevated type 1 immune response in vitiligo (75, 76). Several type 1 IFN, regulated in part by TYK2, have also been implicated in vitiligo pathogenesis (77).

Unlike other JAK family members, which are ubiquitously expressed and associated with many receptor chains, JAK3 is primarily found in hematopoietic cells and is associated with the γc receptor. This receptor is shared by 6 cytokines, including IL-2, IL-4, IL-7, IL-9, IL-15, and IL-21 (64–66).

In vitiligo, IFN-γ and other vitiligo pathogenesis-associated cytokines, including IL-2 and IL-15, drive increased chemokine production, recruitment of melanocyte-reactive immune cells, reduced melanocyte adhesion, enhanced melanocyte apoptosis, and disease reactivation through the JAK/STAT pathway and TEC pathways (63, 78, 79).

In vitiligo, CD4^+^ and CD8^+^ T-cells induce the expression of cytokines associated with γc receptor chains (**Figure 1**). The IL-2 family cytokines, consisting of IL-2, IL-7, and IL-15, while not directly affecting melanocytes or keratinocytes, subsequently activate CD49a^+^/CD8^+^ tissue-resident memory T cells. These cytokines influence multiple aspects of tissue-resident memory T cell behavior, promoting melanocyte apoptosis and maintaining disease activity (46, 47, 58, 79, 80). IL-2, produced by CD4^+^ T-cells, stimulates CD8^+^ tissue-resident memory T cell proliferation (81, 82). As discussed earlier, IL-7 and IL-15 activate and maintain tissue-resident memory T cells (42, 58).

Outside of T cell activation, JAK3 also plays a role in other immune system functions, including NK cells and ILCs. Through activation of *IL-2* expression, JAK3 can lead to higher levels of IFN-γ and NK-cell expansion (with constitutional *JAK3* expression playing a role in certain oncogenic events) (83). Through IL-15, STAT5 signaling leads to maturation of NK cells and maintenance of higher levels of NK cells (84). *Jak3* deficiency in mice blocks ILC differentiation, which may abrogate the secretion of IFN-γ by these cells, leading to induction of melanocyte apoptosis and activation of T-cell melanocyte auto-immunity activation (23, 85).

### 3.2 The TEC family kinase pathways

The TEC family kinase members are cytoplasmic tyrosine kinases involved in many intracellular signaling processes in hematopoietic cells (86–88). The five members include TEC, Bruton’s tyrosine kinase (BTK), IL-2–inducible T-cell kinase (ITK), Resting lymphocyte kinase (RLK/TXK), and Bone-marrow tyrosine kinase gene on chromosome X (BMX) (86–88). *TEC*, *BTK*, and *BMX* are expressed highly in myeloid cells (89), while ITK, RLK, and TEC are important for CD4^+^ and CD8^+^ T-cell development (90).

In vitiligo, ITK (a TEC member), may play a role in maturation of CD8^+^ T-cells (91). APCs when sensing autoantigens take up and process them into autoantigenic peptide:MHC-I complexes. These complexes are presented on the cell surface and recognized by TCRs (24, 36), leading to ITK activation, recruitment to the cell surface, and subsequent T-cell maturation, differentiation, and proliferation (**Figure 1**). Additionally, inhibition of ITK has been shown *in vitro* to disrupt CD8^+^ T-cell-induced IFN-γ secretion (92). In Itk knockout mice, Th2 and T helper type 17 (Th17) cell differentiation and functioning are impacted, leading to dysregulation of regulatory T-cells (Tregs) (93). Also via IFN-γ secretion, ITK plays a role in NK cell activity (94–96). These NK cells secrete cytokines such as IFN-γ, contributing to further immune recruitment.

The involvement of the TCR in recognition of autoantigenic peptide:MHC-I complexes suggests that TEC kinase family members, such as ITK, contribute to the development of vitiligo. This is supported by evidence implicating a role for ITK in other T-cell-mediated autoimmune disorders, including rheumatoid arthritis, psoriasis, and atopic dermatitis (91). RLK and TEC also act downstream of TCR, and may affect IFN-γ secretion from NK cells. RLK, alongside ITK, mediates T-cell and NK cell activation and proliferation (94, 95). T cells that are deficient in ITK and RLK show little expression of *IL-2*, and following activation show a reduction in proliferation (97). In some cases, RLK and ITK also induce expression of “natural killer T-cells”, which express characteristics of both T-cells and NK cells, and secrete IFN-γ in large quantities rapidly (94). Disruption of RLK and ITK also disrupts Th1 and Th2 cell signaling, respectively (98). Increased *RLK* expression is also found in patients with the inflammatory disorder Behcet disease, which is characterized by increased inflammation and Th1 cytokines (99). However, RLK and ITK may serve similar functions in the immune system. In some instances, selective disruption of *RLK* expression was found to have little effect on immune system regulation unless *ITK* expression was also disrupted (95, 100). RLK may function somewhat independently in chemokine receptor signaling, as in murine models, Itk-/Rlk-deficient T cells showed defective responses to various chemokines (101). Collectively, this supports a role for ITK and RLK in vitiligo pathogenesis, with ITK contributing via IFN-γ signaling and RLK contributing via chemokine-induction of Th1 signaling alone, and through IL-2 induced activation of resident memory T cells in combination.

TEC, expressed at lower levels than ITK, BTK, or BMX in T- and B-cells, also shares a role with ITK and BTK, with Tec knockout mice showing no defects in lymphocyte functioning (102). However, it is upregulated at T-cell activation following stimulation with anti-CD3/CD28, and in Th1 and Th2 cells following stimulation with IL-4, IL-12, and IFN-γ, and induces activation of nuclear factor of activated T cell transcription factors (103). This suggests that it may increase the sensitivity of effector or resident memory T-cells.

Additionally, BTK, ITK, RLK, and TEC are expressed in mast cells, which serve as effectors for allergic responses, and innate and adaptive immune responses (104). In mast cells, BTK is thought to be involved in c-kit specific signaling, with other TEC family kinase members potentially serving redundant or regulatory roles (104). BTK, through IL-15-activated STAT5 signaling, also plays a role in the development, maturation, and function of B-cells, CD8^+^ T cells, and NK cells (105–108). *BTK* expression, when disrupted, also reduces expression of *Ifn-γ* in animal models (108). Emerging evidence suggests that BMX may contribute to the pathogenesis of inflammatory disorders (such as vitiligo) through TNF-α and IL-1β signaling to IL-8 (109).

Dual JAK3 and TEC family kinase inhibition with ritlecitinib was shown to decrease certain biomarkers associated with vitiligo pathogenesis in blood samples taken from patients with NSV, including NK and T-cell activation (NCR1, IL-2, IL-15), Th1 activation (TNFSR10B, CXCR3, CCL5, IL-23a, IL-12b), and oxidative stress (NOS3) (76). Other biomarkers expressed in the skin affected include those related to Th2, Th17, and Th22 activation (including *IL-13*, CCL13, CCL18, *IL-17a*, and *IL-22*), and regulatory markers such as CTLA4 and PD1 (76).

Collectively, these studies highlight the importance of both JAK3 and the TEC family kinases in immune cell-signaling and vitiligo pathogenesis and suggest that targeting of these pathways may be a viable therapy option for patients with vitiligo.

## 4 Emerging therapies that target the JAK/STAT pathways and TEC family kinases

Perhaps stemming from its complex pathogenesis, vitiligo has an unpredictable clinical course (110). Common treatment modalities for vitiligo are similar for those used in other autoinflammatory diseases and include topical and oral corticosteroids and topical calcineurin inhibitors, as well as phototherapy (in the form of UV light) to stimulate differentiation and proliferation of melanocyte stem cells, and to provide immunomodulatory effects (1, 48, 111–118). Immunomodulating agents such as methotrexate, cyclosporine, azathioprine, and minocycline are less commonly used and their efficacy and safety have been understudied. Additionally, consensus guidelines do not recommend the use of biologics (118).

Recent improvements in understanding the immunopathogenesis of vitiligo as discussed above have led to the development of new treatments (119). These treatments target key molecules involved in vitiligo pathogenesis. In this section, we will briefly discuss treatments both approved and at various stages of development that target JAK or TEC family kinase members, as well review lessons and insights from other autoimmune conditions and reflect on how these might affect vitiligo treatment development.

### 4.1 Therapies approved for the treatment of vitiligo

Ruxolitinib cream, a topical JAK1/2 inhibitor previously approved for the treatment of atopic dermatitis, was approved by the FDA to treat NSV in July 2022 and by the EC in April 2023 for the treatment of patients with NSV including facial involvement aged ≥12 years (116, 120–122); however, two phase 3 clinical trials (NCT04052425 and NCT04057573) limited topical application to ≤10% of the body surface area (120–122).

### 4.2 Therapies under investigation for the treatment of vitiligo

Other targeted therapies are currently under development for the treatment of vitiligo (**Table 2**). Ritlecitinib is an oral, selective dual inhibitor of JAK3 and the TEC family kinases and is approved to treat severe alopecia areata (123). Through blocking the adenosine triphosphate binding site on these proteins, ritlecitinib irreversibly and selectively inhibits JAK3 and the TEC family kinases (92). In cellular settings, ritlecitinib inhibits signaling of the JAK3-dependent common γ-chain cytokines (IL-2, IL-4, IL-7, IL-9, IL-15 and IL-21). It has high selectivity over the other three JAK isoforms (JAK1, JAK2, and TYK2) as well as over the broader human kinome (65, 92).

**Table 2:** Emerging and approved therapies targeting key molecules involved in vitiligo pathogenesis.

Molecule	Mechanism of action	Method of application	Population treatment approved for or under investigation in	Development status
Ruxolitinib	JAK1/2 inhibition	Topical	Individuals ≥12 years with NSV and facial involvement	Approved July 2022 (USA) and April 2023 (EU) (121, 122)
Ritlecitinib	JAK3/TEC family kinase inhibition	Oral	Individuals ≥12 years with NSV (NCT05583526, NCT06163326) or adults with NSV (NCT06072183)	Phase 3, ongoing (NCT05583526, NCT06072183, NCT06163326)
Upadacitinib	JAK inhibition, favoring JAK1 inhibition	Oral	Individuals ≥12 years with NSV	Phase 3, ongoing (NCT06118411)
Povorcitinib	JAK1 inhibition	Oral	Adults with NSV	Phase 3, ongoing (NCT06113445, NCT06113471)
Deucravacitinib	TYK2 inhibition	Oral	Adults with NSV	Phase 2, recruiting (NCT06327321)
Baricitinib	JAK1/2 inhibition	Oral	Adults with NSV	Phase 2, completed (NCT04822584)
SYHX1901	JAK kinases	Oral	Adults with NSV	Phase 2, recruiting (NCT06511739)
AMG 714	IL-15 inhibition	Intravenous	Adults with vitiligo	Phase 2a, completed (NCT04338581)
TEV-53408	IL-15 inhibition	Subcutaneous	Adults with vitiligo	Phase 1b, recruiting (NCT06625177)
Anifrolumab	Type I interferon inhibitor	Intravenous	Adults with nonsegmental progressive vitiligo	Phase 2, ongoing (NCT05917561)

NSV, nonsegmental vitiligo.

Ritlecitinib is also under investigation for the systemic treatment of NSV (124). In a phase 2b clinical trial (NCT03715829), ritlecitinib demonstrated significant improvement on the centrally read Facial Vitiligo Area Scoring Index (crF-VASI) at Week 24 in patients with active NSV at 50 mg daily with or without a loading dose (100 or 200 mg daily for 4 weeks), with further improvement in the crF-VASI observed up to Week 48 (124). A substudy of the phase 2b trial found that treatment with ritlecitinib significantly reduced biomarkers associated with immune signaling and inflammation in skin and blood of patients with active NSV, and upregulated melanocyte-specific markers; this correlated with clinical improvements (76). Three phase 3 clinical trials assessing ritlecitinib treatment for vitiligo in adults and adolescents aged ≥12 years are ongoing (NCT05583526, NCT06072183 [assessing adults only], and NCT06163326).

Upadacitinib is an oral JAK1 inhibitor and is approved to treat rheumatoid arthritis, atopic dermatitis, ulcerative colitis, psoriatic arthritis, ankylosing spondylitis, and Crohn’s disease (125, 126). In a phase 2 clinical trial (NCT04927975), upadacitinib monotherapy led to repigmentation of facial and body lesions, with significant improvement on the F-VASI and total-VASI (T-VASI) at Week 24 in adults with NSV at 11 mg or 22 mg daily, with further improvement observed up to Week 52 (127). A phase 3 clinical trial in adults and adolescents is ongoing (NCT06118411).

Povorcitinib, an oral selective JAK1 inhibitor, is under investigation for treatment of hidradenitis suppurativa, vitiligo, prurigo nodularis, asthma, and chronic spontaneous urticaria (128). In a phase 2b clinical trial (NCT04818346), once-daily 15 mg, 45 mg, and 75 mg povorcitinib demonstrated significant improvement in T-VASI in patients with NSV at Week 24, with continued improvement up to Week 52 (129). Two phase 3 clinical trials (NCT06113445 and NCT06113471) in adults are ongoing.

Baricitinib, an oral JAK1/2 inhibitor, is approved to treat rheumatoid arthritis, atopic dermatitis, and alopecia areata (130). In a prospective randomized phase 2 clinical trial (NCT04822584), baricitinib combined with narrowband UVB has demonstrated significant superior improvement in F-VASI and T-VASI compared to placebo and narrowband UVB in patients with active NSV at Week 36 (131).

Deucravacitinib, an oral TYK2 inhibitor that inhibits IFN type 1 responses and IL-12 amongst others, is approved to treat plaque psoriasis (75). It is being investigated in an ongoing phase 2 clinical trial (NCT06327321) in patients with NSV.

Other treatments targeting cytokines associated with the JAK/STAT pathway under investigation as treatments for vitiligo include SYHX1901, an oral pan-JAK inhibitor (which is currently recruiting for a phase 2 clinical trial [NCT06511739] in adults with NSV), the intravenous IL-15 inhibitor AMG 714 (currently being investigated in a completed phase 2a clinical trial [NCT04338581] in adults with vitiligo), the subcutaneous IL-15 inhibitor TEV-53408 (currently recruiting for a phase 1b clinical trial [NCT06625177] in adults with vitiligo), and anifrolumab, an intravenous type I interferon inhibitor (currently being investigated in an ongoing phase 2 clinical trial [NCT05917561] in adults with nonsegmental progressive vitiligo).

Several of the treatments discussed above have also seen approvals in other autoimmune inflammatory dermatological diseases, such as alopecia areata and atopic dermatitis, which share some overlapping pathogenesis with vitiligo, particularly in activation of the JAK/STAT pathway (17, 66, 132–140).

### 4.3 Evidence gaps and insights from other autoimmune disease indications

As is the case for autoimmune diseases such as alopecia areata or atopic dermatitis, vitiligo may require long-term treatment due to the underlying pathology and unpredictable course of the disease. As new treatments for vitiligo targeting the JAK/STAT and TEC family kinase pathways are developed and evaluated, the need to address knowledge gaps also arises.

Of note, potential safety concerns associated with long-term usage should be addressed specifically within the vitiligo treatment population. Based on studies of JAK inhibitors in patients with rheumatoid arthritis, the FDA and other regulatory bodies have issued warnings that JAK inhibition may be associated with increased risks of infection, malignancies, thromboembolic events, and lipid levels, and changes in hematologic parameters (141–146). However, differences between those autoimmune skin diseases (such as vitiligo, alopecia areata, or atopic dermatitis), and those with other autoimmune diseases, such as rheumatoid arthritis, including differences in clinical manifestations, underlying pathophysiology, associated comorbidities and risk of adverse events, concomitant treatments, and age, as well as the specific kinases being targeted, may change the safety profile of the JAK inhibitor. Notably, in two phase 3 clinical trials of patients 12 years and older spanning 52 weeks, treatment with topical ruxolitinib cream was not associated with any serious treatment-emergent adverse events (120), and a long-term safety study evaluating ruxolitinib over 104 weeks reported no serious treatment-related adverse events (147). Additionally, short- and long-term systemic JAK and TEC family kinase inhibition has been evaluated in patients with alopecia areata and atopic dermatitis, where these therapies were generally well tolerated and not associated with increased rates of adverse events such as infections or thromboembolic events compared with the general population of patients with alopecia areata or atopic dermatitis (146, 148–153). Ongoing and future studies evaluating long-term treatment with oral/systemic JAK or TEC family kinase inhibition will shed further light on the safety profile specifically in patients with vitiligo.

Studies evaluating long-term treatment efficacy in individuals with vitiligo who receive targeted therapies will also provide evidence on other knowledge gaps currently lacking, such as durability, while evaluations of patients outside of clinical trials will allow health care providers to understand the impact of stopping and restarting treatments.

## 5 Summary

To summarize, recent evidence suggests that the JAK/STAT and TEC family kinase pathways play key roles in vitiligo pathogenesis and disease maintenance. There are currently no targeted systemic treatments approved for the treatment of vitiligo; however, consequently, advances in understanding how these pathways impact pathogenesis has led to the emergence of new targeted therapies for the treatment of vitiligo. As data on long-term safety and efficacy with these targeted treatments emerges, they could have the potential to define a new era of targeted treatments for patients with vitiligo.
